# Association between the number of teeth and all-cause mortality rate in the MASHAD Cohort Study

**DOI:** 10.34172/joddd.025.41913

**Published:** 2025-06-30

**Authors:** Sara Saffar Soflaei, Reza Ekrad-Ferezghi, Behnood Najjari, Mohammad Mobasheri, Seyed Mohammad Reza Mousavi, Arash Pourdad, Mohsen Moohebati, Gordon A. Ferns, Javad Sarabadani, Habibollah Esmaily, Majid Ghayour Mobarhan

**Affiliations:** ^1^Metabolic Syndrome Research Center, Mashhad University of Medical Sciences, Mashhad, Iran; ^2^International UNESCO Center for Health-Related Basic Sciences and Human Nutrition, Mashhad University of Medical Sciences, Mashhad, Iran; ^3^Faculty of Medicine, Birjand University of Medical Sciences, Birjand, Iran; ^4^Faculty of Medicine, Mashhad University of Medical Sciences, Mashhad, Iran.; ^5^Faculty of Paramedicine, Mashhad University of Medical Sciences, Mashhad, Iran; ^6^Heart and Vascular Research Center, Mashhad University of Medical Sciences, Mashhad, Iran; ^7^Department of Cardiology, Faculty of Medicine, Mashhad University of Medical Sciences, Mashhad, Iran; ^8^Brighton and Sussex Medical School, Division of Medical Education, Brighton, UK; ^9^Department of Oral Medicine, Faculty of Dentistry and Dental Research Center, Mashhad University of Medical Sciences, Mashhad, Iran; ^10^Department of Biostatistics, School of Health, Mashhad University of Medical Sciences, Mashhad, Iran; ^11^Social Determinants of Health Research Center, Mashhad University of Medical Sciences, Mashhad, Iran

**Keywords:** All-cause, Cohort studies, Mortality, Tooth

## Abstract

**Background.:**

Previous studies have reported inconsistent results on the relationship between the number of teeth and all-cause mortality. There are several confounding factors in this relationship, especially age. We investigated the relationship between the number of teeth and all-cause mortality among residents in Mashhad, Iran.

**Methods.:**

Of 9704 participants of the Mashhad Stroke and Heart Atherosclerotic Disorder (MASHAD) study conducted in this cohort study, 395 participants were randomly recruited for dental examination. Baseline characteristics, including age, sex, and status of marriage, employment, and education, were collected for all the participants. The number of teeth was recorded by a dentist who also undertook a full dental examination. Individuals were followed up every three years, over 10 years, for the incidence of death. Data were analyzed using SPSS 20, and a *P* value of<0.05 was considered significant. The effect of confounders was reduced using multivariate logistic regression.

**Results.:**

Among 387 eligible participants, the mean age was 48.60±8.24 years, and most were female. The number of teeth was significantly related to age (*P*<0.001), marital status (*P*=0.002), and educational attainment (*P*=0.001). Over ten years of follow-up, 15 of the participants died. Among baseline variables, only age was significantly associated with death (*P*=0.008). The number of teeth was significantly associated with all-cause mortality after adjustment for age, sex and marital status, employment, and educational attainment (*P*=0.003, OR=0.926, 95% CI: 0.880‒0.974).

**Conclusion.:**

Number of teeth is an independent predictor of all-cause mortality, especially in older individuals.

## Introduction

 Teeth play a vital role in human physiology, with important functions such as chewing, swallowing, speaking, facial aesthetics, and social interactions.^1^ Severe caries, chronic periodontitis, and tooth loss account for 2% of the overall burden of human diseases worldwide.^2^ In 2015, over 3.5 billion individuals worldwide were affected by oral diseases such as dental caries, periodontal disease, and tooth loss.^3^ This widespread issue of poor oral health is a significant concern for public health, as evidenced by the estimated 42% of US adults with periodontitis and 11% who have lost all their natural teeth within five years.^4^ Previous studies have revealed an association between inadequate oral health and all-cause mortality, including cancer and cardiovascular mortality.^5,6^ The number of lost teeth is considered a crude factor of poor oral health.^7^ Therefore, a key objective in oral health is to enhance the percentage of people who have functional teeth.^8^ Periodontal disease, often caused by dental plaque,^9^ is one of the main causes of tooth loss.^10,11^ A significant relationship has been established between periodontal diseases and cardiovascular diseases (CVDs),^12^ diabetes, pneumonia,^13^ as well as increased healthcare expenses.^14,15^ Periodontal disease is widely recognized as a risk factor for malignant diseases due to its association with the systemic inflammatory response.^16^ The presence of disease and tooth loss can negatively affect dietary and nutritional intake, ultimately compromising overall systemic health.^17^

 Previous studies have had a substantial variation in the experimental design, and it is not possible to eliminate the possibility of remaining confounding factors.^9,18-20^ Furthermore, some of the analyzed articles contained samples with an average age predominantly exceeding 60 years, which ultimately rendered the findings unreliable.^17,21-28^ While previous studies in Iran, such as the Golestan Cohort Study,^29^ have linked poor oral health to increased mortality in economically transitioning populations, there remains a lack of research on this association in large urban centers like Mashhad, which limits the generalizability of existing findings to diverse urban populations and underscores the need for locally relevant data to inform public health strategies. Given Mashhad’s diverse population, varying access to healthcare, and public health relevance, it offers a unique setting to examine the link between tooth loss and mortality within an urban Iranian population. To our knowledge, this is the first study to investigate this association, specifically in the Mashhad population.

 The importance of oral health is evident in today’s society. Over the last few years, the number of teeth, as one of the main indicators of oral health, has received increased attention for its connection with systemic diseases, severity, and mortality. This study aims to evaluate the association between the number of natural teeth and all-cause mortality.

## Methods

###  Study population

 This study was conducted on a sub-sample of the Mashhad Stroke and Heart Atherosclerotic Disorder (MASHAD) cohort study.^30^ This study was initiated in 2010 to explore the risk factors of CVDs among the 35‒65-year-old citizens of Mashhad, the second largest city in Iran. Using a stratified cluster random sampling, 9704 individuals were included in the study. Data on demographics, anthropometrics, and lifestyle were collected for all participants. Inclusion criteria for the sub-sample were participants aged 35–65 from the MASHAD Cohort who provided informed consent and had complete data on demographic and clinical variables. Exclusion criteria were loss to follow-up because of lack of contact number or migration and incomplete dental examination. Considering the mortality rate of 5% in the study population, the mean ± SD of teeth in Yu et al’s study^12^ and the following formula, the sample size was calculated at n = 18 for each group.


$$N=\frac{{\left(Z_{1-\alpha /2}+Z_{1-\beta }\right)}^{2}\times (S_{1}^{2}+S_{2}^{2})}{{\left(X_{1}-X_{2}\right)}^{2}}$$

Assuming a 95% confidence level, 80% power, and an effect size estimated from prior studies to ensure representativeness and sufficient power for subgroup analysis of the larger cohort, 395 individuals were selected using proportional stratified random sampling based on key demographic variables such as age, sex, and residential region within MASHAD study participants. The selected individuals were visited by dentists, and the number of their teeth was recorded. Dental examinations were conducted by trained dentists using a standardized protocol, as tooth counting was performed through visual inspection under appropriate lighting, with only erupted, non-extracted natural teeth included in the count. Inter-examiner reliability was assessed through calibration sessions before data collection, and a subset of participants was re-examined by different dentists to ensure consistency. Participants whose dental examination was not complete were excluded from the analysis. Potential sources of bias were the exclusion of dietary habits and denture use, which could influence tooth count and associated health outcomes. Dietary factors, such as sugar intake and the use of dentures, may affect oral health; however, they were not directly assessed in this study. These exclusions may limit the generalizability of the results, as individuals with significant dietary variation or denture use may differ in tooth preservation. However, the impact of these factors is likely minimized due to the study’s focus on natural tooth count and the overall population being relatively homogeneous in these respects.

###  Study follow-up

**Figure 1:**
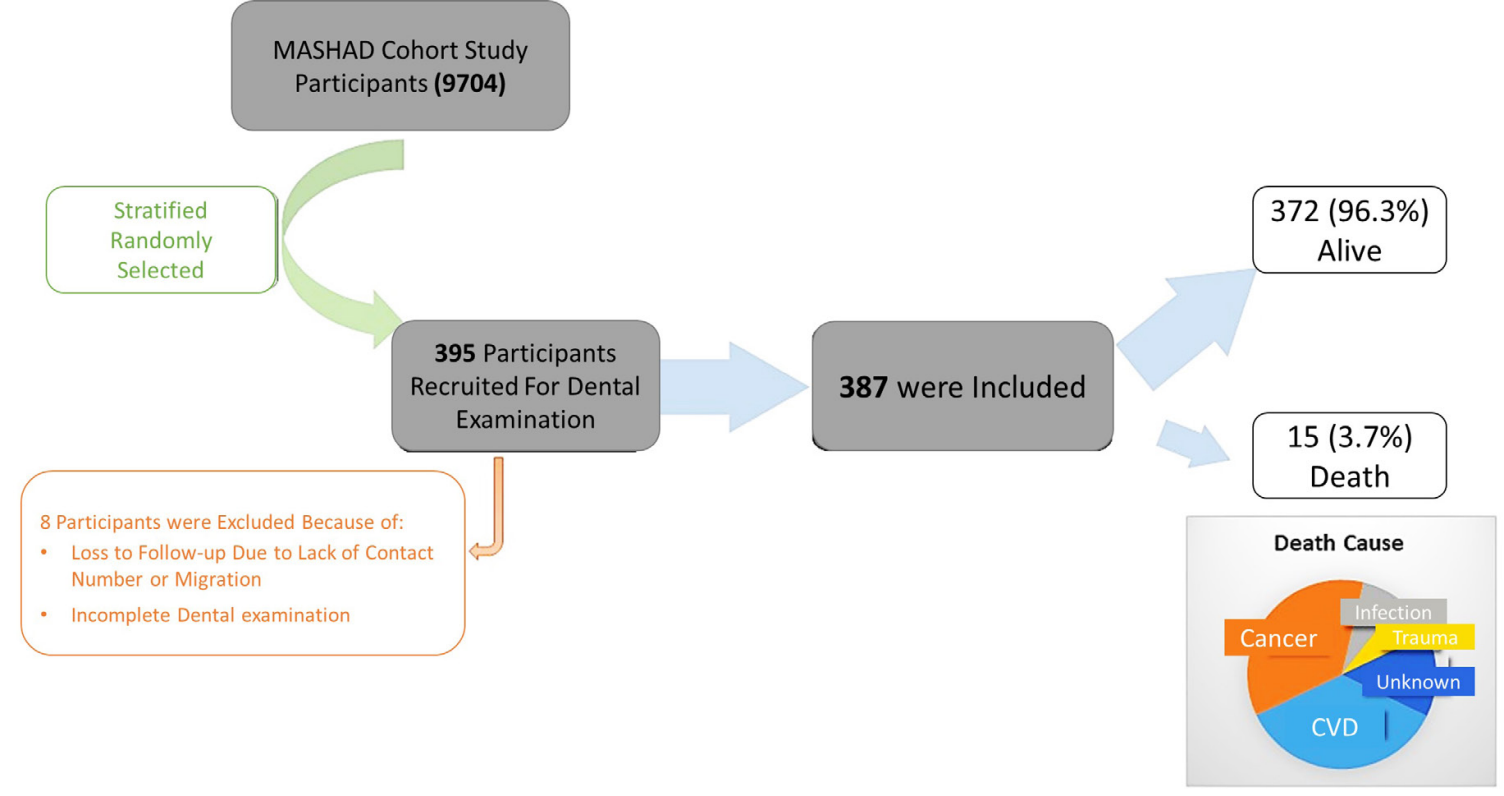
Flow diagram showing the recruitment and number of deaths of study participants

 Eligible participants who had data about the number of teeth available were followed up every three years over 10 years. Participants who died were recorded as the target of the current study. The cause of death was extracted from the death registry of the Iranian Ministry of Health and Education. During the study, seven individuals dropped out of the target population due to a lack of contact number or having moved to a new city (Figure 1). A comparison of baseline characteristics between dropouts and those included in the analysis showed no significant differences, suggesting minimal risk of attrition bias. However, the small number of dropouts further reduces the likelihood of any meaningful impact on the study’s overall findings.

###  Statistical methods

 The Kolmogorov-Smirnov (K-S) test was used to assess normality parameters. Mann-Whitney, chi-squared, and logistic regression were also used for statistical purposes. SPSS 20 (IBM Corp., 2011) was used for statistical calculations (*P* < 0.05). Additionally, COX regression was used to reduce the effect of confounders. Association between the number of existing teeth and all-cause mortality was reported using a hazard ratio (HR) with a 95% confidence interval (CI). A log rank test was also applied, and Kaplan-Meier survival plots were provided to show the significance.

## Results

 This study comprised a sample of 387 people with a mean age of 48.60 ± 8.24 years, with 223 (57.2%) females. Most of the population did not have a career or a high level of education. Additionally, most participants were married (Table 1). The participants had a mean of 18.08 teeth (SD = 8.98). The results indicated a significant correlation between the number of teeth and age, marital status, and education level. Over 10 years of follow-up, 15 out of 387 participants had died. The causes of death were cancer (n = 5), CVD (n = 5), infection (n = 1) and trauma (n = 1). Two were due to an unknown cause. The mean ± SD of the number of teeth in dead and alive cases was 18.40 ± 8.81 and 10.27 ± 9.98, respectively (*P* = 0.007).

**Table 1:** Baseline characteristics according to tooth number and all-cause mortality

**Variables**	**Tooth number**			**Mortality**		
	**Mean (SD)**	**Median (1**^st^**-3**^rd^** quartile)**	***P*** **valu**^a^	**Alive (n=372)**	**Death (n=15)**	***P*** **value**^b^
Age (y)			**<0.001**			**0.001**
≤ 49	20.93 (7.28)	23 (18-26)		212 (57.0%)	2 (13.3%)	
> 49	14.56 (9.63)	17 (6-22)		160 (43.0%)	13 (86.7%)	
Sex			0.065			0.535
Male	17.33 (9.02)	20 (12-25)		158 (42.5%)	6 (40.0%)	
Female	18.64 (8.93)	21 (16-25)		214 (57.5%)	9 (60.0%)	
Job status			0.118			0.264
Employment	18.80 (8.77)	22 (15-25)		137 (36.8%)	3 (20.0%)	
Unemployment	18.11 (8.94)	21 (15-25)		193 (51.9%)	11 (73.3%)	
Retired	15.60 (9.59)	19 (9-24)		42 (11.3%)	1 (6.7%)	
Marital status			**0.013**			0.222
Single	13.78 (10.07)	16 (3-25)		21 (5.6%)	2 (13.3%)	
Married	18.35 (18.85)	21 (15-25)		351(94.4%)	13 (86.7%)	
Education			**0.002**			0.180
Low	17.57 (9.15)	21 (13-25)		332 (89.2%)	15 (100%)	
High	22.53 (5.77)	24.5 (20-27)		40 (10.8%)	0 (0%)	

^a^ Analyzed by Mann-Whitney test. ^b^ Analyzed by chi-square test. Bold Numbers show statistically significant difference.

**Figure 2:**
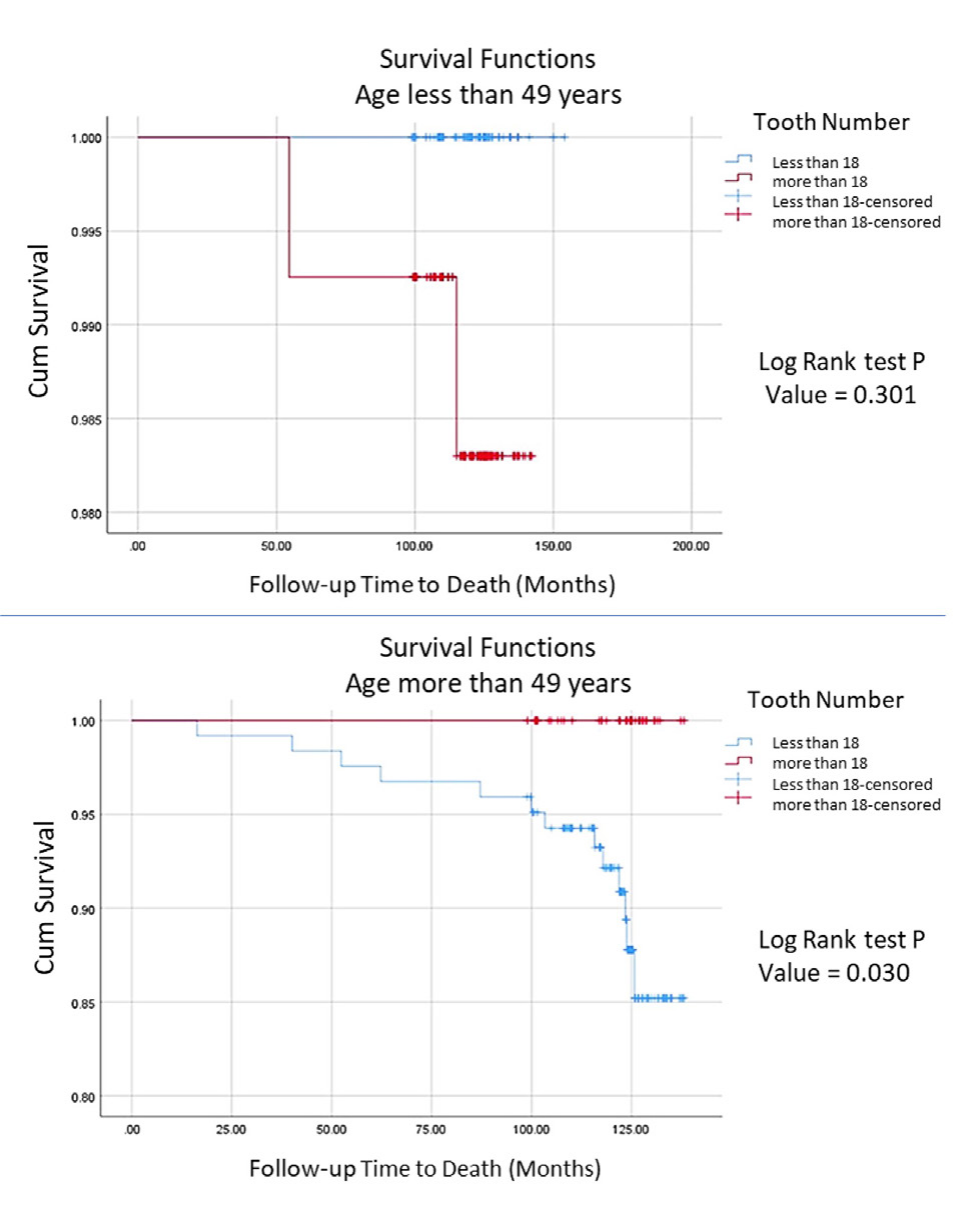
Cumulative death plots according to age groups: (above) age < 49; (below) age > 49

 Based on the presented information, in participants > 49 years of age, the risk of all-cause mortality decreased by 6.1% for each additional tooth. Also, at all ages, the risk of all-cause mortality decreased by 8.4% for every tooth count (Table 2). Figure 2 indicates the cumulative deaths through follow-up time. The number of teeth was significantly associated with all-cause mortality in participants over 49 years (*P* = 0.030).

**Table 2:** Association of tooth number and all-cause mortality

**Groups**	**Hazard ratio**	**95% CI **	***P*** ** value**
Total^1^	0.926	0.880-0.974	0.003
Age ≤ 49^2^	1.100	0.777-1.558	0.591
Age > 49^2^	0.939	0.886-0.997	0.038

Analyzed by logistic regression ^1^ Adjusted for all variables in Table 1. ^2^ Adjusted for all variables in Table 1 except age.

## Discussion

 The number of lost teeth has been proposed as a basic indicator of dental disease.^19^ The primary causes of tooth loss are dental caries and periodontitis,^20^ accounting for 2% of the global burden of human diseases. Fewer teeth have been linked to various systemic diseases, especially atherosclerotic CVDs.^31^ The process conducted in this study discovered a potential link between tooth loss and mortality. The cohort study involved 387 participants between the ages of 35 and 65. The findings showed that people > 49 years of age had a 10% reduction in the risk of all-cause mortality for each additional tooth. Additionally, at all ages, there was a 7% reduction in overall mortality risk for every tooth count. Our research also indicated that the number of teeth was significantly associated with age, marital status, and education, but no consistent associations were found with sex and job status.

 Previous studies have also reported positive correlations between poor oral health and increased mortality rates.^32^ For example, in a cohort study of 50,023 participants in Golestan Province, Iran, an increased risk of death was observed in both unadjusted and adjusted analyses for all-cause mortality and cause-specific mortality, including CVDs, cancer, and injuries. Furthermore, this study also discusses the effects of dentures and tooth brushing frequency on the mortality rate.^29^

 The Finnish Parogene Cohort, a subset of the Finnish Corogene Cohort, consists of 508 adults with a mean age of 63.3 ± 9.1. It also confirms the effect of the number of teeth on all-cause mortality, with a record of 69 deaths during the follow-up, mainly due to CVD, neoplasms, diabetes, and diseases of different body systems. In this study, in addition to the Golestan Cohort, it was observed that the patients who died were often men.^29,33^

 A study in Taiwan showed that losing 11‒20 or more than 20 teeth, particularly in underweight elderly individuals (age ≥ 65 years), significantly increased the risk of all-cause mortality compared to participants with 0‒10 teeth lost. It is worth noting that the results are not entirely relatable due to the participants’ old age.^27^ In another study conducted in the same year in Japan on 569 participants aged 70 years at baseline, similar results were concluded during a 5-year follow-up period: having 19 teeth or fewer increased the risk of death by almost 2.5 times compared to having 20 teeth or more.^25^ A newer study in 2023 in China, conducted on 5,403 participants aged 65 ± 10.47 years old, showed an even more significant relation between the number of teeth and mortality. The study showed that compared to having 20 or more teeth, subjects with 10‒19 teeth had an increase in mortality rate by more than twice, and for subjects with 0‒10 teeth, the risk increased up to nearly four times in 3.1 ± 1.3 years of follow-up.^22^

 In a study on 84,160 adults aged 42 ± 13 years in 2020, the number of teeth was shown to be associated with all-cause, cancer-related, and cardiovascular mortality. In this paper, the main mechanism studied was the connection between masticatory capacity, nutrient intake, and mortality rate. The most notable effect of missing teeth in this study was on CVD.^19^

 The studies mentioned above were mainly about all-cause mortality, but some have focused on specific-cause mortality rates. A study in 2019 aimed to find the relationship between tooth loss and long-term cardiovascular outcomes, including myocardial infarction (MI), ischemic stroke, and heart failure (HF). This study was conducted on 4,404,970 subjects with a 7.59 ± 0.72 year follow-up. Out of this population, 1.5% died during the follow-up period, and 1.9% of the rest of the subjects suffered from one of the previously mentioned cardiovascular events. In general, the incidence rate of these events was above 0.65 per 1000 person-years. Based on the analysis, the highest risk belonged to the participants with > 15 lost teeth.^34^

 Cancer is also a significant factor in mortality associated with tooth loss. In 2015, a retrospective cohort study was conducted on 1,385 residents of Shanghai Ninth People’s Hospital aged 75 years and older, which recorded 31 deaths during a 4-year follow-up. Almost a quarter of the casualties were due to cancer. Therefore, this study concluded that there is a notable connection between the number of teeth and oral cancer mortality. It is worth noting that this study also considered alveolar bone loss as a second factor affecting mortality, which slightly compromises the usability of the results for our study.^28^ On the contrary, a Japanese study conducted on 20,445 dentists with a mean follow-up of 9.5 years concluded that although frequent tooth brushing could decrease the risk of upper aerodigestive tract (UADT) cancer, the number of teeth did not have a significant impact on cancer and, consequently, on the mortality rate. One of the reasons for this discrepancy is the bias in the study population, as dentists’ higher knowledge of oral health can influence the course of the study, resulting in a different conclusion than previous studies.

 Although the link between tooth loss and mortality is consistent, the mechanism is still unclear. Several possibilities are being discussed; the most prominent theory is the negative effect of tooth loss on masticatory function. This can decrease dietary diversity,^22^ which mainly leads to a low-fiber and antioxidant diet due to a lack of fruit and vegetable consumption.^19^ Another reason may be the increased burden of inflammatory agents.^35^ Although our findings align with prior research as discussed, caution is warranted when generalizing these results to other regions, particularly those with differing socioeconomic, dietary, and healthcare contexts.

 The present study had limitations due to its small sample size, resulting in decreased statistical power for the analyses. However, our long follow-up period and repetition of examinations helped compensate for this limitation to a large extent. Another limitation is that we did not consider the use of dentures or implants and their potential effect on the mortality rate. Additionally, the participants’ dietary habits were not considered in our study. Despite adjusting our analyses for baseline patient characteristics, it is possible that common chronic and undetected subclinical diseases or unmeasured diseases could have contributed to reduced survival. Moreover, although only seven individuals dropped out of the study, leading to a relatively small number of exclusions, potential selection bias cannot be completely ruled out. The individuals who did not participate in follow-up assessments may differ systematically from those who remained, particularly regarding health status, dental care practices, or socioeconomic factors. While the small number of dropouts likely reduces the impact of this bias, and although efforts were made to minimize this bias, it is still possible that their exclusion could influence the observed association between the number of teeth and mortality. Additionally, despite adjusting for baseline patient characteristics, residual confounding from unmeasured variables, such as systemic inflammation, cannot be ruled out. Inflammation has been shown to play a key role in both oral health and systemic diseases, and its impact on mortality could not be fully captured in this study.

 This study has several strengths, including its extensive and multiple follow-up periods of up to 10 years with 3-year intervals. Additionally, the analysis was conducted on a relatively middle-aged population (48.60 ± 8.24 years) compared to other studies involving elderly and community-dwelling subjects. This choice helps to reduce false positives associated with age-related confounding factors.

###  Practical Implications

 Our findings suggest that maintaining a higher number of natural teeth may be associated with reduced mortality risk. This underscores the importance of oral health in overall well-being. Public health initiatives should consider integrating oral health assessments into routine medical check-ups, particularly for middle-aged populations, to facilitate early detection and intervention. Additionally, promoting oral hygiene practices and access to dental care could play a crucial role in enhancing longevity, as a study by Fukuhara et al^36^ found that lower educational attainment and depressive symptoms were associated with increased tooth loss, highlighting the need for targeted interventions addressing these factors to improve oral health outcomes.

## Conclusion

 We found that the number of teeth was significantly associated with the mortality rate in our study population, indicating that improving and maintaining adequate oral health might substantially increase longevity, which highlights the clinical and public health importance of preserving natural teeth, as tooth loss may contribute to mortality through mechanisms such as poor nutrition, systemic inflammation, and social vulnerability. Most of our participants had a low educational level, and all the casualties in our study belonged to this group. This may suggest a close yet indirect connection between educational level and the mortality rate. Additionally, it is worth mentioning that more than two-thirds of our recorded deaths were due to CVD and cancer. However, we did not investigate the specific relationship between these causes of mortality. We recommend that future studies explore the association between the number of teeth and these diseases.

## Competing Interests

 There is no competing interest.

## Ethical Approval

 The study protocol was approved by the Ethics Committee of Mashhad University of Medical Sciences (IR.MUMS.Medical.Rec.1386.250), and written informed consent was obtained from all participants.

## References

[1] Sischo L, Broder HL (2011). Oral health-related quality of life: what, why, how, and future implications. J Dent Res.

[2] GBD 2015 DALYs and HALE Collaborators (2016). Global, regional, and national disability-adjusted life-years (DALYs) for 315 diseases and injuries and healthy life expectancy (HALE), 1990-2015: a systematic analysis for the Global Burden of Disease Study 2015. Lancet.

[3] Liu J, Zong X, Vogtmann E, Cao C, James AS, Chan AT (2022). Tooth count, untreated caries and mortality in US adults: a population-based cohort study. Int J Epidemiol.

[4] Wu Z, O’Brien KM, Lawrence KG, Han Y, Weinberg CR, Sandler DP (2021). Associations of periodontal disease and tooth loss with all-cause and cause-specific mortality in the Sister Study. J Clin Periodontol.

[5] Adolph M, Darnaud C, Thomas F, Pannier B, Danchin N, Batty GD (2017). Oral health in relation to all-cause mortality: the IPC cohort study. Sci Rep.

[6] Schwahn C, Polzer I, Haring R, Dörr M, Wallaschofski H, Kocher T (2013). Missing, unreplaced teeth and risk of all-cause and cardiovascular mortality. Int J Cardiol.

[7] El Osta N, Hennequin M, Tubert-Jeannin S, Abboud Naaman NB, El Osta L, Geahchan N (2014). The pertinence of oral health indicators in nutritional studies in the elderly. Clin Nutr.

[8] Hobdell M, Petersen PE, Clarkson J, Johnson N (2003). Global goals for oral health 2020. Int Dent J.

[9] Tsukamoto M, Naito M, Wakai K, Naito T, Kojima M, Umemura O (2021). Tooth brushing, tooth loss, and risk of upper aerodigestive tract cancer: a cohort study of Japanese dentisits. Nagoya J Med Sci.

[10] Löe H, Anerud A, Boysen H, Morrison E (1986). Natural history of periodontal disease in man Rapid, moderate and no loss of attachment in Sri Lankan laborers 14 to 46 years of age. J Clin Periodontol.

[11] Salvi GE, Mischler DC, Schmidlin K, Matuliene G, Pjetursson BE, Brägger U (2014). Risk factors associated with the longevity of multi-rooted teeth Long-term outcomes after active and supportive periodontal therapy. J Clin Periodontol.

[12] Yu YH, Chasman DI, Buring JE, Rose L, Ridker PM (2015). Cardiovascular risks associated with incident and prevalent periodontal disease. J Clin Periodontol.

[13] Suma S, Naito M, Wakai K, Naito T, Kojima M, Umemura O (2018). Tooth loss and pneumonia mortality: a cohort study of Japanese dentists. PLoS One.

[14] Choi SE, Sima C, Pandya A (2020). Impact of treating oral disease on preventing vascular diseases: a model-based cost-effectiveness analysis of periodontal treatment among patients with type 2 diabetes. Diabetes Care.

[15] Shin JH, Takada D, Kunisawa S, Imanaka Y (2021). Effects of periodontal management for patients with type 2 diabetes on healthcare expenditure, hospitalization and worsening of diabetes: an observational study using medical, dental and pharmacy claims data in Japan. J Clin Periodontol.

[16] Ishikawa S, Konta T, Susa S, Ishizawa K, Togashi H, Ueno Y (2020). Association between presence of 20 or more natural teeth and all-cause, cancer-related, and cardiovascular disease-related mortality: Yamagata (Takahata) prospective observational study. BMC Oral Health.

[17] Qi L, Qian Y, Zhu F, Cao N, Lu H, Zhang L (2020). Association between periodontal disease and tooth loss and mortality in an elderly Chinese population. Aging Clin Exp Res.

[18] Koka S, Gupta A (2018). Association between missing tooth count and mortality: a systematic review. J Prosthodont Res.

[19] Darnaud C, Thomas F, Danchin N, Boutouyrie P, Bouchard P (2020). Masticatory capacity and mortality: the preventive and clinical investigation center (IPC) cohort study. J Dent Res.

[20] Liljestrand JM, Havulinna AS, Paju S, Männistö S, Salomaa V, Pussinen PJ (2015). Missing teeth predict incident cardiovascular events, diabetes, and death. J Dent Res.

[21] Furuta M, Takeuchi K, Adachi M, Kinoshita T, Eshima N, Akifusa S (2018). Tooth loss, swallowing dysfunction and mortality in Japanese older adults receiving home care services. Geriatr Gerontol Int.

[22] Dai M, Song Q, Lin T, Huang X, Xie Y, Wang X (2023). Tooth loss, denture use, and all-cause and cause-specific mortality in older adults: a community cohort study. Front Public Health.

[23] Yun JH, Ki SK, Kim J, Chon D, Shin SY, Lee Y (2020). Relationships between cognitive function and frailty in older Korean adults: the moderating effect of the number of teeth. Arch Gerontol Geriatr.

[24] Tanaka T, Takahashi K, Hirano H, Kikutani T, Watanabe Y, Ohara Y (2018). Oral frailty as a risk factor for physical frailty and mortality in community-dwelling elderly. J Gerontol A Biol Sci Med Sci.

[25] Hirotomi T, Yoshihara A, Ogawa H, Miyazaki H (2015). Number of teeth and 5-year mortality in an elderly population. Community Dent Oral Epidemiol.

[26] Caplan DJ, Ghazal TS, Cowen HJ, Oliveira DC (2017). Dental status as a predictor of mortality among nursing facility residents in eastern Iowa. Gerodontology.

[27] Hu HY, Lee YL, Lin SY, Chou YC, Chung D, Huang N (2015). Association between tooth loss, body mass index, and all-cause mortality among elderly patients in Taiwan. Medicine (Baltimore).

[28] Qian Y, Yu H, Yuan W, Wu J, Xu Q, Mei N (2020). Alveolar bone loss, tooth loss and oral cancer mortality in older patients: a retrospective cohort study. Clin Interv Aging.

[29] Vogtmann E, Etemadi A, Kamangar F, Islami F, Roshandel G, Poustchi H (2017). Oral health and mortality in the Golestan Cohort Study. Int J Epidemiol.

[30] Ghayour-Mobarhan M, Moohebati M, Esmaily H, Ebrahimi M, Parizadeh SM, Heidari-Bakavoli AR (2015). Mashhad stroke and heart atherosclerotic disorder (MASHAD) study: design, baseline characteristics and 10-year cardiovascular risk estimation. Int J Public Health.

[31] Beukers N, Su N, Loos BG, van der Heijden G (2021). Lower number of teeth is related to higher risks for ACVD and death-systematic review and meta-analyses of survival data. Front Cardiovasc Med.

[32] Polzer I, Schwahn C, Völzke H, Mundt T, Biffar R (2012). The association of tooth loss with all-cause and circulatory mortality Is there a benefit of replaced teeth? A systematic review and meta-analysis. Clin Oral Investig.

[33] Liljestrand JM, Salminen A, Lahdentausta L, Paju S, Mäntylä P, Buhlin K (2021). Association between dental factors and mortality. Int Endod J.

[34] Lee HJ, Choi EK, Park JB, Han KD, Oh S (2019). Tooth loss predicts myocardial infarction, heart failure, stroke, and death. J Dent Res.

[35] Kotronia E, Wannamethee SG, Papacosta AO, Whincup PH, Lennon LT, Visser M (2021). Poor oral health and inflammatory, hemostatic, and cardiac biomarkers in older age: results from two studies in the UK and USA. J Gerontol A Biol Sci Med Sci.

[36] Fukuhara S, Asai K, Kakeno A, Umebachi C, Yamanaka S, Watanabe T (2021). Association of education and depressive symptoms with tooth loss. J Dent Res.

